# Effectiveness Regarding Hantavirus Detection in Rodent Tissue Samples and Urine

**DOI:** 10.3390/v13040570

**Published:** 2021-03-29

**Authors:** Mónika Madai, Győző Horváth, Róbert Herczeg, Balázs Somogyi, Brigitta Zana, Fanni Földes, Gábor Kemenesi, Kornélia Kurucz, Henrietta Papp, Safia Zeghbib, Ferenc Jakab

**Affiliations:** 1 National Laboratory of Virology, BSL-4 Laboratory, Szentágothai Research Centre, University of Pécs, H-7624 Pécs, Hungary; somogyi.balazs@pte.hu (B.S.); brigitta.zana@gmail.com (B.Z.); fanni4444@gmail.com (F.F.); kemenesi.gabor@gmail.com (G.K.); phencsi@gmail.com (H.P.); zeghbib.safia@gmail.com (S.Z.); 2 Institute of Biology, Faculty of Sciences, University of Pécs, H-7624 Pécs, Hungary; hgypte@gamma.ttk.pte.hu (G.H.); kornelia.kurucz@gmail.com (K.K.); 3 Bioinformatics Research Group, Szentágothai Research Centre, University of Pécs, H-7624 Pécs, Hungary; herczeg.robert@pte.hu

**Keywords:** naturally infected, hantavirus detection, urine, rodent, tissue

## Abstract

The natural hosts of Orthohantaviruses are rodents, soricomorphs and bats, and it is well known that they may cause serious or even fatal diseases among humans worldwide. The virus is persistent among animals and it is shed via urine, saliva and feces throughout the entirety of their lives. We aim to identify the effectiveness of hantavirus detection in rodent tissue samples and urine originating from naturally infected rodents. Initially, animals were trapped at five distinct locations throughout the Transdanubian region in Hungary. Lung, liver, kidney and urine samples were obtained from 163 deceased animals. All organs and urine were tested using nested reverse transcription polymerase chain reaction (nRT-PCR). Furthermore, sera were examined for IgG antibodies against Dobrava–Belgrade virus (DOBV) and Puumala virus (PUUV) by Western blot assay. IgG antibodies against hantaviruses and/or nucleic acid were detected in 25 (15.3%) cases. Among Apodemus, Myodes, and Microtus rodent species, DOBV, PUUV and Tula virus (TULV) were clearly identified. Amid the PCR-positive samples, the nucleic acid of the viruses was detected most effectively in the kidney (100%), while only 55% of screened lung tissues were positive. Interestingly, only three out of 20 rodent urine samples were positive when tested using nRT-PCR. Moreover, five rodents were seropositive without detectable virus nucleic acid in any of the tested organs.

## 1. Introduction

Orthohantaviruses (*Hantaviridae* family) are negative-sense, single-stranded RNA viruses with three genome segments, including the small (S) segment (encodes the nucleoprotein), medium (M) segment (encodes glycoproteins) and large (L) segment, which encodes the RNA-dependent RNA polymerase [1]. Orthohantaviruses may cause serious or even fatal diseases, such as hemorrhagic fever with renal syndrome (HFRS), caused by Hantaan (HNTV), Dobrava–Belgrade (DOBV) and Seoul (SEOV) viruses, while Puumala virus (PUUV) is the etiological agent that causes nephropathia epidemica (NE). The mortality rates of HFRS range from 1 to 15% and are dependent on the causative agents [2]. In contrast, PUUV is responsible for more than 9000 infections annually, throughout Europe, with a significantly lower-case fatality rate of 0.1–0.4%. Among New World orthohantaviruses, the Sin Nombre (SNV) and Andes orthohantaviruses (ANDV) cause hantavirus cardiopulmonary syndrome (HCPS) with an average case fatality rate at or near 40% [3,4,5]. Globally, 150,000–200,000 human cases of orthohantavirus infections are reported annually [6]. Hantaviruses are transmitted to humans by persistently infected rodents, soricomorphs and bats indirectly via inhalation of the aerosolized excreta of infected animals or directly through a rodent bite [7]. In Europe, the two major human pathogenic orthohantaviruses are DOBV, carried by the yellow-necked mouse (*Apodemus flavicollis*), the striped field mouse (*Apodemus agrarius*), and the wood mouse (*Apodemus sylvaticus*), and PUUV, which is carried by the bank vole (*Myodes glareolus*) [1,8,9]. From a varied perspective, Tula virus (TULV) can be found in Europe, but the human pathogenic nature intrinsic to its species is debated [10,11]. In consideration of their natural animal hosts, these viruses do not cause disease, despite their influential characteristics upon the host’s survival and the fact that they cause histopathological changes regarding infected tissues [4,12,13]. Virus infection induces a life-long IgG antibody response after 2–3 weeks in small mammals. However, the life-long presence of these viruses in tissues and excreta is questionable.

In this study, we aimed to identify the most suitable tissue in the detection of hantavirus. For this reason, various rodent tissues and urine originating from naturally infected animals were tested by molecular detection methods. Additionally, we also investigated whether naturally infected rodents are able to transmit the virus over a lengthy period of time via their urine, as formerly hypothesized [14,15].

## 2. Materials and Methods 

### 2.1. Sample Collection

Rodents were trapped as part of an ecological research project at five different locations throughout the Southern Transdanubian Region between 2012 and 2015, from March to October. Live rodent traps were used with quadrat sampling patterns in each trapping period. Every month, five-night standard capture periods occurred. The traps were checked once/twice per day depending on the trap location. 

Deceased animals acquired from live traps were used in our study. After the species, sex and weight determination, rodents were frozen and stored (−80 °C) until dissection. During autopsy, internal organs, such as the lung, liver and kidney, were removed. Urine was taken directly from the bladder using a syringe when available. All samples were stored at −80 °C until further analysis. 

### 2.2. Extraction of Nucleic Acid, PCR Amplification, Sequencing

Nearly 50 mg of lung, liver and kidney tissue samples were homogenized in 500 µL of phosphate-buffered saline (1 × PBS) using Minilys homogenizer (Bertin Instruments, France) with one glass bead (2.5–2.8 mm). Afterwards, viral nucleic acid was extracted from 200 µL of tissue supernatant or urine using Viral Nucleic Acid Extraction Kit II (Geneaid, Xinbei, Taiwan) in accordance with the manufacturer’s recommendations. The RNA yield was quantified using NanoDrop™ (Thermo Scientific, Waltham, MA, USA). The RNA samples were stored at −80 °C until analysis.

In regard to hantavirus RNA detection, we used nested reverse transcription polymerase chain reaction (nRT-PCR), following the method described by Klempa and his colleagues. Firstly, specific degenerated primers (HAN-L-F1: 5′-ATGTAYGTBAGTGCWGATGC-3′ and HAN-L-R1: 5′-AACCADTCWGTYCCRTCATC-3′) were used; then, the nested PCR portion was made with HAN-L-F2: 5′-TGCWGATGCHACIAARTGGTC-3′ and HAN-L-R2: 5′-GCRTCRTCWGARTGRTGDGCAA-3′ primers [16]. The reaction was performed using a QIAGEN OneStep RT-PCR Kit (Qiagen, Hilden, Germany) with the following conditions: at 50 °C for 30 min, followed by an initial denaturation at 95 °C for 15 min, and then 40 cycles of amplification (each cycle included a denaturation step at 94 °C for 1 min, an annealing step at 53 °C for 30 s, an extension step at 72 °C for 1 min) and a final elongation at 72 °C for 10 min. In total, 2 µL of the first round PCR products were amplified with inner primers under the following conditions: initial denaturation at 95 °C for 5 min, followed by 40 cycles of amplification (denaturation at 94 °C for 1 min, annealing at 52 °C for 45 s and extension at 72 °C for 1 min) and a final elongation at 72 °C for 10 min. Second round PCR products were visualized by agarose gel electrophoresis in 2% agarose gel stained with GR Green (Labgene Scientific, Châtel-Saint-Denis, Switzerland).

The amplicons from positive samples were purified by a Gel/PCR DNA Fragments Kit (Geneaid) and bi-directionally sequenced with a BigDye Terminator v1.1 Cycle Sequencing Kit (Applied Biosystems™, Foster City, CA, USA, CA) on an ABI Prism 310 DNA Sequencer (Applied Biosystems™). Nucleic acid sequences were identified by GenBank BLAST searches based on the most significant homology.

### 2.3. Serological Screening by Western Blot Analysis

Rodent blood samples were screened for the presence of IgG antibodies against DOBV and PUUV by Western blot (WB) analysis. We applied recombinant DOBV and PUUV antigens, both produced in an *Escherichia coli* bacterial expression system, as previously described [17,18]. Nucleocapsid proteins (PUUV and DOBV) were loaded into the wells of Mini-PROTEAN^®^ TGX^™^ Precast Gels (Bio-Rad Laboratories, Irvine, CA, USA). Following electrophoresis, proteins were transferred to 0.45 µm pore size nitrocellulose membranes (Bio-Rad Laboratories) using a Trans-Blot^®^ SD Semi-Dry Transfer Cell (Bio-Rad Laboratories), at 0.12 A for 30 min. The membranes were painted with Ponceau S (Sigma-Aldrich, St. Louis, MO, USA), which made the proteins visible. We used 5% non-fat dry milk for blocking (Blotting-Grade Blocker, Bio-Rad Laboratories) in Tris-buffered saline (TBS) (pH = 7.5) for one hour. Rodent blood samples were diluted 1:100 in TBS (pH = 7.5) containing 0.1% bovine serum albumin (BSA) (Sigma-Aldrich) and 0.05% Tween^®^ 20 (Sigma-Aldrich) and membranes were incubated for 30 min at room temperature. Following incubation, membranes were rinsed in 0.05% TBS–Tween^®^ 20 (TBS–T) for 3 × 10 min. Horseradish peroxidase-conjugated rabbit anti-mouse IgG (Dako, Glostrup Kommune, Denmark) was used as a secondary antibody, diluted 1:800 in TBS–T containing 0.05% BSA. Next, an incubation period was performed for 30 min at room temperature. Membranes were rinsed for 10 min three times using TBS–T and once (10 min) with TBS. Development was carried out using 3,3′-diaminobenzidine (DAB) (Bio-Rad Laboratories) in TBS in accordance with the manufacturer’s recommendations.

## 3. Results

In our study, we examined various tissues and urine samples originating from 163 out of 665 trapped and perished rodents (because urine samples were available only from 163 animals). The trapping was part of an ecological study with a catch number of 25,183. Rodents were categorized into seven different species: 22 (13.5%) *A. agrarius*, 64 (39.2%) *A. flavicollis*, 6 (3.7%) *A. sylvaticus*, 53 (32.5%) *M. glareolus*, 6 (3.7%) field voles (*Microtus agrestis*), 11 (6.7%) common voles (*Microtus arvalis*) and 1 (0.6%) European water vole (*Arvicola amphibious*). Out of a total of 163 rodents, hantavirus nucleic acid and/or IgG antibodies against hantaviruses were detectable in 25 cases (15.3%). In consideration of these rodents, 19 belonged to the Apodemus species, four to the Microtus species and two were *Myodes glareolus*. There were 20 hantavirus-positive samples acquired using nRT-PCR from at least one of the investigated organs (lung, liver and kidney). Among these 20 nRT-PCR rodent samples that tested positive, the kidney tissues were positive in each rodent (20/20; 100%), while the fewest PCR-positive samples originated from lung tissue (11/20; 55%). It is very likely the virus is present in the urine for only a brief period of time since only three urine samples were positive for hantaviruses (Table 1).

In reflecting upon the serological investigations, antibodies against hantaviruses in the sera were detected in 18 cases out of 21 hantavirus-positive samples. In five cases, nucleic acid could not be detected in any tested organs; however, IgG antibodies were present in the sera, meaning that these rodents were exclusively seropositive. The presence of a maternal antibody can be ruled out since these individuals were adults. In another 13 cases, both hantavirus nucleic acid and IgG antibodies against hantaviruses were present. Due to the lack of a detection assay, Microtus voles were not tested by any serological test.

A clear connection was not found between virus detection in the lungs and seropositivity. In two cases, negative serological results were obtained even when the virus was clearly detectable by nRT-PCR in the lung tissue. In contrast, 13 animals with a negative nRT-PCR result obtained from the lungs were seropositive. In the case of liver and kidney tissues, a different detection rate was observed. For the liver and kidneys, the number of nRT-PCR-positive and serology-negative animals was three for both tissues, while the number of nRT-PCR-positive and serology-negative rodents was six for the liver and five for the kidneys. Unfortunately, we could not determine when the infection occurred and how it developed over time since our examination was based on samples collected strictly from deceased animals.

Based on the sequencing data, we identified DOBV in Apodemus mice, PUUV in *Myodes glareolus* and TULV in Microtus voles, with the greatest homologies of 100%, 95% and 89%, respectively.

## 4. Discussion

Hantavirus infections are considered persistent in rodents. A number of studies illustrate how hantaviruses are present in various types of tissues and excreta (saliva, urine and feces) [14,19,20,21,22,23]. Few studies have focused on naturally infected rodents, thereby substantiating the premise that there are differences in virus shedding between naturally and laboratory-infected rodents [14]. We examined naturally infected, deceased animals collected from box traps. For years, researchers thought the most appropriate tissues regarding hantavirus detection was limited to the lungs [22,23,24,25]. In our study, out of 20 PCR-positive lung tissue samples, only 11 (55%) were positive for hantaviruses, while 19 liver and all kidney tissue samples demonstrated viral nucleic acid positivity. Yanagihara et al. and Gavrilovskaya et al. found that PUUV infection was persistent among rodents; therefore, the virus antigen was detected in lung tissues for nearly a year. In contrast, the virus was undetectable in the kidneys [23,24,25,26]. Our research shows contrasting results regarding the case of DOBV in Apodemus mice; however, in the case of PUUV, we could not make a determination since two *Myodes glareolus* samples were positive. As a result, further investigations are necessary. Lee et al. investigated Hantaan virus (HNTV)-infected *Apodemus agrarius,* which were able to infect their cage mates via urine and saliva [22]. However, the detection of the virus in the kidney did not result in its detection in the urine. Due to the low number of positive samples, this premise may be considerably murky and additional in vivo studies are required. Presumably, among Apodemus mice, the host immune system can eliminate the virus among host rodents [27,28,29]. These differences were highlighted in a review authored by Meyer and Schmaljohn in which PUUV antigen persistence in lung tissues was detectable for nearly a year, while HNTV antigens were detectable for just 14 days [21]. These data support the possibility that, in hosts that cause HFRS in humans, the virus can be detectable in the kidney for a long time after infection, while, in species that cause HCPS, the virus can be present in the lungs for a long time. HFRS symptoms include serious renal failure in humans, while, in the case of HCPS, cardiac and respiratory problems are common. If this were the case, Puumala and Tula viruses could be the exceptions, as they cause only mild or asymptomatic disease and require further investigation in naturally infected rodents. It has been demonstrated in a previous study conducted by Easterbrook and Klein and Németh et al. that IgG serology is negative in the beginning of hantavirus infection, whereas PCR tests are positive [17,29]. Importantly, this was observed in a few cases, in accordance with our results (14%). Therefore, as far as we are concerned, due to the high cost of molecular biology methods, it can be useful to first use serological methods for screening and monitoring animals and then, in the case of seropositive rodents, select the liver or, more preferably, the kidneys for molecular biological detection. It is clearly visible from our examinations that, in the case of Apodemus species, both organs (liver and kidney) have better detectability rates of the virus than the lungs, which have been used in several surveys so far [22,24].

In this study, we could not confirm whether lifelong virus shedding occurs via the urine. The most appropriate tissue regarding hantavirus detection by PCR methods is the rodent kidneys; however, if there are detectable virus particles in the kidneys, we cannot be absolutely certain whether these are also present in the urine. Based on our results, further long-term experiments could be performed on naturally infected wild rodents in order to gain more knowledge about the detailed nature of this viral infection.

## Figures and Tables

**Table 1:** Detailed molecular biological results of various organs and urine samples originating from rodents (nRT-PCR) along with the Western blot (WB) serological results and hantavirus species resulting from sequencing results (Abbreviations: AAG: Apodemus agrarius, AFL: Apodemus flavicollis, MAR: Microtus arvalis, MAG: Microtus agrestis, MGL: Myodes glareolus, nt.: not tested by serology, * only seropositive rodents).

	PCR-Positive Rodents						
	Rodent Species	Lung	Liver	Kidney	Urine	Serology (IgG)	Virus Species
		(nRT-PCR)				(WB)	
1	AAG	Pos	Pos	Pos	-	-	*Dobrava–Belgrade*
2	AAG	-	Pos	Pos	Pos	-	
3	AAG	Pos	Pos	Pos	-	Pos	
4	AAG	Pos	Pos	Pos	-	Pos	
5	AAG	-	Pos	Pos	-	Pos	
6	AAG	-	Pos	Pos	-	Pos	
7	AAG	-	Pos	Pos	-	Pos	
8	AAG	-	Pos	Pos	-	Pos	
9	AAG	-	-	Pos	-	Pos	
10	AFL	Pos	Pos	Pos	-	-	*Dobrava–Belgrade*
11	AFL	Pos	Pos	Pos	-	Pos	
12	AFL	Pos	Pos	Pos	-	Pos	
13	AFL	Pos	Pos	Pos	-	Pos	
14	AFL	-	Pos	Pos	-	Pos	
15	AFL	-	Pos	Pos	-	Pos	
16	MAG	Pos	Pos	Pos	Pos	*nt.*	*Tula*
17	MAR	Pos	Pos	Pos	-	*nt.*	*Tula*
18	MAR	Pos	Pos	Pos	-	*nt.*	
19	MAR	Pos	Pos	Pos	Pos	*nt.*	
20	MGL	-	Pos	Pos	-	Pos	*Puumala*
**Total**		**11/20**	**19/20**	**20/20**	**3/20**	**13/16**	
**Seropositive Rodents**							
21	AFL	-	-	-	-	Pos *	*Dobrava–Belgrade*
22	AFL	-	-	-	-	Pos *	
23	AFL	-	-	-	-	Pos *	
24	AFL	-	-	-	-	Pos *	
25	MGL	-	-	-	-	Pos *	*Puumala*
	**Total**	**0/5**	**0/5**	**0/5**	**0/5**	**5/5**	

## Data Availability

Publicly available datasets were analyzed in this study.
